# A Review on Anti-Tumor Mechanisms of Coumarins

**DOI:** 10.3389/fonc.2020.592853

**Published:** 2020-12-04

**Authors:** Yi Wu, Jing Xu, Yiting Liu, Yiyu Zeng, Guojun Wu

**Affiliations:** ^1^School of Stomatology, Central South University, Changsha, China; ^2^Department of Microbiology, School of Basic Medical Science, Central South University, Changsha, China

**Keywords:** anticancer agents, carbonic anhydrase inhibitors, caspase-dependent apoptosis, coumarin, reactive oxygen species

## Abstract

Coumarins are a class of compound with benzopyrone as their basic structure. Due to abundant sources, easy synthesis, and various pharmacological activities, coumarins have attracted extensive attention from researchers. In particular, coumarins have very significant anti-tumor abilities and a variety of anti-tumor mechanisms, including inhibition of carbonic anhydrase, targeting PI3K/Akt/mTOR signaling pathways, inducing cell apoptosis protein activation, inhibition of tumor multidrug resistance, inhibition of microtubule polymerization, regulating the reactive oxygen species, and inhibition of tumor angiogenesis, etc. This review focuses on the mechanisms and the research progress of coumarins against cancers in recent years.

## Introduction

**Figure 1:**
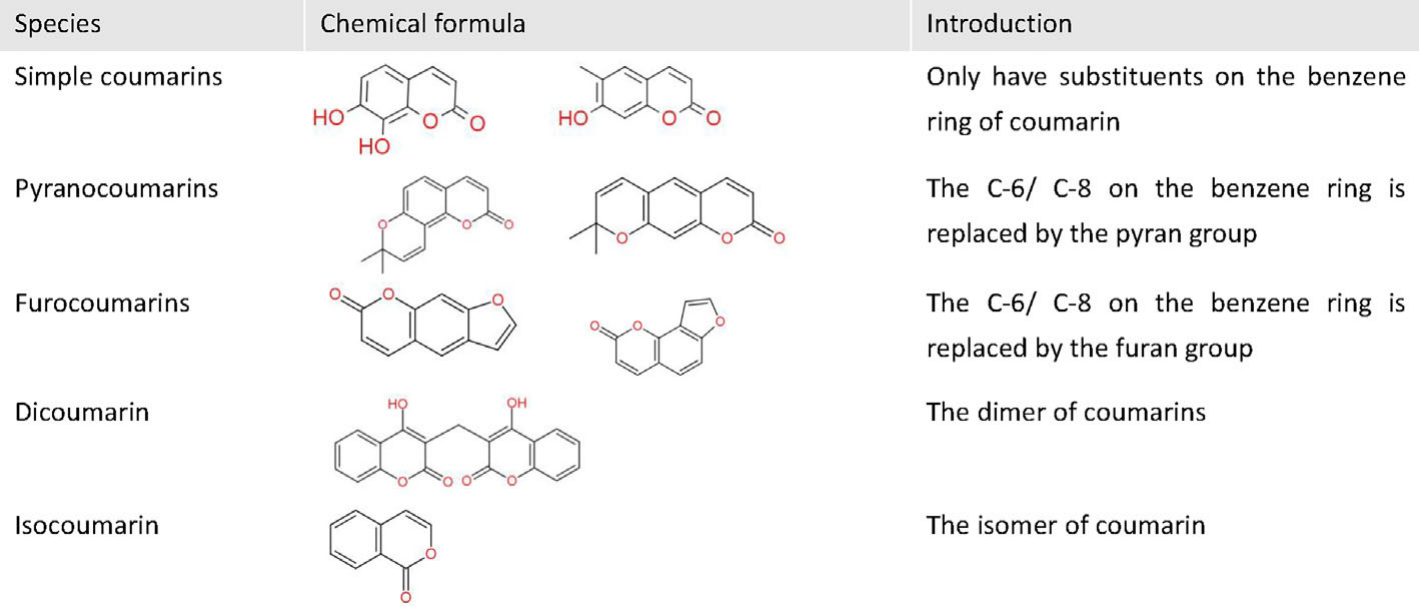
The basic classification of coumarins. This figure shows some chemical formulas and basic introduction of five classes coumarins.

Cancer is the second leading cause of death worldwide after cardiovascular disease, and a major threat to human health. Coumarins are a class of natural compounds widely found in a variety of plant families, including Umbelliferae, Compositae, Leguminosae, Rutaceae, mulberry, mignonette, and thyme (1). They have a wide range of pharmacological activities, such as anti-inflammatory, anticoagulant, antibacterial, antifungal, antiviral, anticancer, anti-hypertensive, etc (2). Moreover, coumarin is characterized by a simple structure, benzopyrone, on which there are multiple substitution sites. According to the different substituents, coumarins can be divided into five classes: simple coumarins, pyranocoumarins, furocoumarins, dicoumarin, and isocoumarin (**Figure 1**). By modifying the structure of coumarin and introducing functional groups, researchers have synthesized more complex and diverse coumarin derivatives with more application value and more performance. In recent years, coumarin and its derivatives have shown an extremely wide and inestimable potential in the field of anti-tumor therapy. Their anti-tumor mechanisms are very diverse, including inhibiting carbonic anhydrase (CA), targeting PI3K/Akt/mTOR signaling pathway, inhibiting multiple drug resistance (MDR), and inducing apoptosis, etc. In this review, the anti-tumor mechanisms and the research progress of coumarins have been emphasized in recent years.

## Anticancer Mechanisms

### Coumarins Act as CA Inhibitors

CA is a zinc-containing metal enzyme, catalyzing reversible hydration of carbon dioxide, maintaining pH inside and outside the cell, and transmembrane transport of ions, etc., and affecting a variety of metabolic activities (3). There are 16 different CA subtypes at least, among which CA I and CA II belong to a cytoplasmic enzyme and CA IX and CA XII belong to transmembrane protease. Tumor cells and their microenvironment are often hypoxic and increased glycolysis to meet increased metabolic needs, leading to a large accumulation of lactic acid. The expression of CA IX and CA XII in tumor cells is strongly induced by hypoxia-inducible transcription factor (HIF). The two types of carbonic anhydrase can catalyze the reversible hydration of carbon dioxide, convert it into bicarbonate ions and hydrogen ions, and expel hydrogen ions out of the cells to make the tumor cells maintain a slightly intracellular alkalinity and an extracellular acidity, which is conducive to the growth and spread of primary tumors and the development of drug resistance, leads to the formation of metastases (3, 4). CA IX and CA XII are overexpressed in various malignant tumors and can be used as important targets for the design of anticancer compounds.

Based on the structure of Coumarin and sulfonamide, Belma (5) designed a series of compounds and tested their abilities to inhibit the expression of CA I, CA II, CA IX, and CA XII in colon cancer cells. They found that these compounds can effectively restrain the CA IX and CA XII expression. 4-((((2-((1-(3-((2-oxo-2h-chromen-7-yl)foxy)propyl)1h-1,2,3-triazol-4-yl)methoxy)naphthalen-1-yl)-methylene)amino) methyl) benzenesulfonamide (8i) exhibited the ability of selective inhibition of cell proliferation *via* specific inhibition of CA IX and CA XII expression in human colon cancer cells with the Ki is 45.5nM and 596.6 nM, respectively. Belma (6) also synthesized 16 kinds of bis-coumarin derivatives (5a-5p) containing three thiazole rings and alkyl chains, and assessed their inhibitory activities against CA I, CA II, CA IX, and CA XII. They found that all synthetic compounds showed selective inhibitory activities against tumor-related CA IX and CA XII in the high nM range. Among them, 4-methyl-7-((1-(12-(2h-(2-oxo-chromen-7-yl)foxy)dodecyl)1h-1,2,3-triazol-4-yl)methoxy)-2h-chromen-2-one (5p) and 4-methyl-7-((1-(1-(2h-(2-oxo-chromen-7-yl) foxy) decyl)1h- 1,2,3-triazol-4-yl)methoxy)-2h-chromen-2-one (5n) showed the highest CA XII and CA IX inhibition with the Ki is 144.6 71.5 nM, respectively.

Some 1-aryl and 2-aryl-substituted Coumarin derivatives showed selective inhibitory activities to CA IX and CA XII (7). Angapelly (8) synthesized a series of 4-sulfamoylphenyl/sulfocoumarin benzamides and evaluated their inhibitory effects on five subtypes of CA. They found that all these sulfocoumarin compounds are weak or invalid inhibitors for CA I and CA II, but can effectively inhibit tumor-related CA IX and CA XII in the high nanomolar to micromolar ranges.

Yu (9) synthesized a series of new dihydroartemisinin-coumarin hybrids. These hybrids showed moderate cytotoxicity to human colon and breast cancer cells. The mechanism included targeting CA IX, resulting in the intracellular accumulation of hydrogen ions, arresting the cell cycle at G0/G1 phase, and a sharp decline in mitochondrial membrane potential, promoting apoptosis and inducing ferroptosis.

In addition, Ferraroni (10) synthesized a series of Coumarins and corresponding 2-thioxocoumarines and tested their inhibitory activities to human carbonic anhydrase CA I, CA II, CA IX, and CA XII, and found that 7-[(1-Phenyl-1H-1,2,3-triazol-4-yl)methoxy]-2H-chromen-2-one (15) and 7-[(1-phenyl-1H-1,2,3-triazol-4-yl)methoxy]-2H-chromene-2-thione (16) was the most effective and the selective inhibitors of CA IX and CA XII, with the Kis are comparable to or slightly lower than the standard sulfonamide AAZ. To explore the inhibitory mechanism of 2-thioxocoumarin to CA, Ferraroni (10) examined the X-ray crystal structure of 6-hydroxy-2-thioxocoumarin which bound to CA II and found that the inhibitory mechanism of 2-thioxocoumarin is quite different from other coumarins, which work as zinc binders or occlusion of the active site entrance, anchor to the metal-ion-coordinated water/hydroxide ion, or bind to outside of the active sites, then hydrolyzed by CA, finally form the corresponding 2- hydroxycinnamic acid derivatives. However, the X-ray crystal structure of CA II- thioxocoumarin adducts showed that the exo-sulfur atom is anchored to the zinc-coordinated water molecule, whereas the scaffold establishing favorable contacts with the amino acid residues from the active sites.

### Coumarins Can Inhibit PI3K/AKT/mTOR Signaling Pathway

PI3K is an intracellular phosphatidylinositol kinase. PI3K can activate a series of protein kinase (PKA, PKC, and PKB) and plays an important role in the cell proliferation, apoptosis, migration, and differentiation. AKT, a serine/threonine kinase, also known as PKB, is one of the key downstream target sites for PI3K, and it is closely related to the cell proliferation and apoptosis. AKT can activate CDK4 and CDK2, regulate cycle-dependent protein inhibitor P27, and enable cells to complete the cell cycle successfully. AKT can also show its antiapoptotic effect through various channels, such as inactivating Bax and caspases family, inhibiting the activity of GSK3, which can accelerate cell apoptosis by degrading cytoskeleton protein β-catenin and inhibiting cell adhesion, activating transcription factor NF-κB and promoting the repair of cellular DNA damage, inhibiting the expression of pro-apoptotic gene FasL, inhibiting the release of cytochrome C and apoptotic factors from mitochondria, and so on. mTOR is also a serine/threonine kinase and is a downstream target site for AKT. Activated mTOR can activate translation inhibition molecule (4E-BP1) and ribosomal protein p70S6K. When 4E-BP1 is phosphorylated, its binding ability to eIF-4E is weakened, and eIF-4E binds to other translation initiation factors to initiate protein translation. Activated p70S6K can also promote protein synthesis. PI3K/AKT/mTOR is a very important signaling pathway in cell survival, growth, metabolism, proliferation, differentiation, metastasis, and cell cycle regulation (11, 12). The signaling pathway is closely related to the pathogenesis of tumors because of often mutation in human tumor cells. Abnormal activation of this pathway plays an important role in tumor development, progression, and drug resistance, thus this pathway is an important target for tumor therapy (13, 14).

Wang (15) synthesized 15 kinds of benzylsulfone coumarin derivatives (5a-5o) and found that 3-[(4-fluorobenzyl)sulfonyl]-6-nitro-2h-chromen-2-one (5h) and 6-bromo-3-[(4-fluorobenzyl)sulfonyl] -2h-chromen-2-one (5m) exibited the strongest inhibitory activities against PI3K, with inhibition rates of 50.3% and 50.8% at 20 μM respectively *in vitro* experiments. In addition, 5h and 5m had broad-spectrum anti-tumor activities, with the IC50 values for 5 tumor cell lines (Hela, HepG2, H1299, hct-116, and McF-7) ranging from (18.1-32.6) μM and (29.3-42.1) μM, respectively. Moreover, 5h and 5m can appropriately embedded into the active sites of PI3Kα and PI3Kβ, and interact with important residues.

5-methoxypsoralen, also known as bergamot and parsley alkali, is a natural product of furocoumarins and usually isolated from psoralen. Guo (16) found that 5-methoxypsoralen inhibits the expression and phosphorylation of PI3K, Akt, and mTOR in human glioma cells, thereby completely down-regulating the expression and activation of the PI3K/Akt/mTOR signaling pathway. 5-methoxypsoralen can also cause DNA damage by fragment the DNA of human glioma cells, and induce the appearance of autophagy vacuoles.

Isofraxidin is a natural Coumarin compound. Shen (17) found that isofraxidin can inhibit the phosphorylation of Akt in human colorectal cancer cells.

Hedyotis diffusa is an ethnic medicine used for anti-cancer treatment in Chinese medicine clinics. The total coumarins of Hedyotis diffusa (TCHD) is a kind of extract with anti-proliferative activity. Jiang (18) tested the apoptosis inductive effect of TCHD on human myelodysplastic syndromes cell line (SKM-1) and found that TCHD could inhibit the expression of PI3K, Akt and the phosphorylation of Akt and P65, thereby inducing NF-κB inactivation.

Imperatorin show anti-tumor properties by inhibiting the phosphorylation of PI3K, Akt, and mTOR in human gastric adenocarcinoma cells (19). Dong (20) synthesized a novel hybrid of 3-benzyl coumarin deca-B-ring derivative and nitric oxide donor benzenesulfonyl furan glycans, and confirmed that this hybrid could inhibit the expression of mTOR and the phosphorylation of mTOR and Akt in non-small-cell lung cancer cells.

Dahong (21) isolated Ferulin C, a natural sesquiterpene coumarin, from Ferula ferulaeoides, and tested its anti-proliferative activity against breast cancer cells *in vitro* and *in vivo*. *In vitro*, by decreasing the expression and nuclear localization of p-AKTSer473, Ferulin C (20μM) significantly inhibited the expression and phosphorylation of AKT and its downstream member mTOR, thus inducing autophagy of tumor cells.

### Coumarins Can Affect Apoptotic Associated Proteins

Caspase-2, -3, -6, -7, -8, -9, and -10 are the key mediators of apoptosis in caspases family. Caspase-2, -8, -9, and -10 are the initiators of apoptosis. After receiving the apoptosis signal, caspase-2 can be self-catalyzed to activate itself, and initiate apoptosis. Caspase-3, -6, and -7 are executers of apoptosis, activated by upstream promoters, can hydrolyze many of the structural and functional proteins, such as poly-ADP-ribose polymerase (PARP) (22). PARP is an important DNA repair enzyme activated by the damaged DNA, identify and bind to the damaged DNA and poly ADP glycosylated DNA repair-related proteins and get involved in the DNA repair process, maintaining the survival and stability of cells. However, when DNA is severely damaged, over-activated PARP will lead to cell death because of the energy exhaustion in the repair process. In addition, poly(ADP-ribose) generated by PARP activation can destroy mitochondria and release the apoptosis-inducing factor. During apoptosis, caspase-3 is activated and PARP is lysed to avoid unnecessary repair of dying cells. Therefore, lysed PARP is considered as an important indicator of caspase-3 activation (23). B cell lymphoma-2 (Bcl-2) is one of the most important oncogenes in apoptosis research. The Bcl-2 family includes pro-apoptotic and anti-apoptotic proteins. Bad, Bax, and PUMA are pro-apoptotic proteins in the Bcl-2 family, which are mainly located in the cytoplasm. Once induced by apoptotic factors, they converge to the outer membrane of mitochondria to form transmembrane channels, which enable mitochondria to release cytochrom C, and activate caspases, thereby promoting apoptosis. Bcl-2 is an anti-apoptotic protein in the Bcl-2 family, which is mainly located on the outer membrane of mitochondria and inhibits cell apoptosis by blocking the release of cytochrome C (22). Coumarin compounds can regulate the expression of these apoptosis-related proteins and achieve the goal of tumor inhibition.

Ferulin C, a natural sesquiterpene coumarin, is extracted from from Ferula ferulaeoides. In breast cancer cells, Ferulin C (20 μM) substantially elevated the expression of Bax, and reduced the expression of Bcl-2. Ferulin C also caused the cleavage of caspase3, caspase7, caspase9, and PARP, which indicated the activation of the classical mitochondria apoptotic pathway (21).

Yao (24) extracted a series of coumarin compounds from Juglans mandshurica with 75% ethanol and tested the cytotoxicity of these compounds to two kinds of hepatocellular carcinoma cell lines *in vitro*. The experiments showed that some compounds had moderate anti-tumor activities to these cell lines. The results of Western blot analysis showed that under the action of xanthyoxylin (compound 2) and 6, 7, 8-trimethoxyl-coumarin (compound 5), the levels of lysed caspase-7 and PARP increased, and the levels of pro-caspase-7 decreased.

When combined with Akt inhibitor MK2206, isofraxidin had a synergistic effect on liver cancer cells, significantly decreasing the expression of anti-apoptotic protein Bcl-2 and increasing the expression of pro-apoptotic proteins, including caspase-3, caspase-9 and Bax (17). In addition, caspase-3, -8, -9, and PARP in SKM-1 were significantly activated by TCHD. The single-stranded DNA bind to the overacted PARP and break it to poly(ADP-ribose), which destroys mitochondria and releases apoptosis-inducing factor (18). Elshemy (25) synthesized three series of coumarin hybrids by hybridizing 8-methoxy coumarin with three bioactive moieties, chalcone 2a-c, acrylohydrazide 4a-c, and pyridine 6a-b and 7a-b. These coumadin hybrids can up-regulate the expression of caspase-3 proteins in HCC cell lines and caspase-3 and caspase-9 proteins in leukemia cell lines, and down-regulate the expression of Bcl-2 and the up-regulation of Bax protein expression levels in HCC and leukemia cell lines.

Nordin (26) isolated a coumarin derivative PulchrinA that connected a long alkyl on the coumarin heterocyclic ring from natural product Enicosanthellum pulchrum for the first time and investigated the ability to initiate apoptosis in human ovarian cancer cells. The results showed that Pulchrin A induced the down-regulation of Bcl-2 protein and the up-regulation of Bax protein by activating caspase-3 and caspase-9, inducing strong cytotoxicity to ovarian cancer cells, and the IC50 value was 22 μM.

Lin (27) synthesized a nitro-coumarin derivate, named 5,7-dimethoxy-4-methyl-6-nitro-chromen-2-one, which up-regulated the expression of BAX and PUMA by cleaving PARP, thereby inducing cytotoxicity in wild-type or KRAS mutated colon cancer cells.

Esculetin, a kind of 6,7-dihydroxyl derivative, can be found in various medicinal plants, such as Cichorium intybus, Artemisia capillaries, Ceratostigma willmottianum, Citrus limonia, etc. Arora (28) treated three kinds of pancreatic cancer cell lines with esculetin, and found that the expression of caspase-3, -8, and -9, as well as the activation level and cleavage form in pancreatic cancer cell lines, were significantly increased. Esculetin can also cause the loss of mitochondrial membrane potential in pancreatic cancer cells and increase the cytoplasmic level of cytochrome C, which in turn mediates the activation of caspase-3 and caspase-9, leading to apoptosis.

Musa (29) synthesized a series of 7,8-Diacetoxy-3-arylcoumarin derivatives (5a-h) and evaluated their cytotoxicity to human prostate (PC-3) and breast cancer (MDA-MB-231) cancer cell lines *in vitro*. 7,8-Diacetoxy-3-(4-methylsulfonyl phenyl) coumarin (5f) is the most active derivative and has the highest cytotoxicity and selectivity to PC-3 cell lines. 5f induced the up-regulated expression of apoptotic proteins such as caspases 3, caspases8, Bid, and Smac/DIABLO, and also induced PC-3 cells apoptosis by reducing mitochondrial membrane potential.

Han (30) synthesized a series of shikonin derivatives and evaluated their anti-human cervical cancer activity. Among them, PMMB232 showed the best anti-proliferative activity, and the IC50 value was 3.25 ± 0.35 μM. PMMB232 induced apoptosis by reducing mitochondrial membrane potential in human cervical cancer cells. PMMB232 also dose-dependently reduced the expression level of PARP and promoted the PARP cleavage.

### MDR Inhibition

MDR refers to the phenomenon of broad-spectrum drug resistance in tumors, an important cause of tumor recurrence. The main mechanism of MDR is that transmembrane proteins such as p-glycoprotein (P-gp) and multidrug resistance-related protein 2 can transfer various anti-tumor drugs from the cytoplasm to the outside of the cells by using the energy from the hydrolysis of ATP. Studies have shown that coumarins can inhibit MDR by inhibiting P-gp or multidrug resistance-related protein 2.

Baghdadi (31) isolated six kinds of coumarin compounds (mansorin-A, mansorin-B, mansorin-C, mansorins-I, mansorin-II, and mansorin-III) from the heartwood of Mansonia gagei family Sterculariaceae, and detected the potential anticancer activities of these compounds against breast cancer, cervical cancer, colorectal cancer, and liver cancer cells. Mansorin-II and mansorin-III showed the best inhibitory effects to these cell lines, with IC50 values ranging from 0.74 µM to 36 µM and 3.95 to 35.3 µM, respectively. In addition, mansorin-II can enhance the anticancer effect of taxol. This synergistic effect may be related to interfering with the efflux activity of P-gp pumps.

Based on high affinity inhibitors, by means of molecular docking simulation and pharmacophore study, Tripathi (32) designed and synthesized a series of new coumarin derivatives, which can dock at active site cavity of P-gps, have a higher binding affinity to the target protein P-gp and can more effectively inhibit the efflux process, thereby enhancing the bioavailability of various anti-tumor drugs and reducing the growth of breast cancer stem cells.

Kasaian isolated and purified 14 sesquiterpene coumarins from the roots of 4 species of Feruar plants, and evaluated the MDR reversal properties of these sesquiterpene coumarins in A2780/RCIS cells (cisplatinum-resistant derivatives of human ovarian cancer cell line A2780P). Some compounds had obvious MDR reversal effects. The combination of nontoxic concentrations of sesquiterpene coumadin (20 µM) with cisplatin significantly enhanced the cytotoxicity of cisplatin on A2780/RCIS cells. The results showed that conferdione and samarcand had the highest inhibitory effects on pump efflux of multidrug resistance-related protein 2 (33).

### Inhibition of Microtubule Polymerization

Microtubules are the main components of the cytoskeleton and play an important role in maintaining cell morphology, cell division and proliferation, and signal material transportation. Clinically, antitumor drugs can inhibit the mitosis of tumor cells by promoting the depolymerization of microtubules or inhibiting their aggregation, so that the tumor cells can be stagnate in the M-phase. There are three drug binding sites on the microtubules, namely colchicine, vincristine, and paclitaxel binding sites. Colchicine, vincristine, and paclitaxel are also commonly used as microtubule inhibitors. In addition, colchicine, vincristine, and paclitaxel are transport substrates for P-gp pumps, so multi-drug resistance of tumor cells may occur.

Cao (34) synthesized a series of new 4-substituted coumarins and tested their anti-proliferation ability against a variety of tumor cells. Among them, 5-chloro-n-(2-methoxy-5-(Methyl (2-oxo-2H-Chromen-4-yl)amino) pentanamide (compound 65) showed strong anti-tumor proliferation ability (IC50 value was 3.5-31.9 nM). Compound 65 disrupted microtubule networks in hepatocellular carcinoma cells in a pattern similar to colchicine, and also induce human ovarian cancer cells to stagnate in the G2/M phase and promote their apoptosis. More importantly, compound 65 showed significant antiproliferative activity to P-gp overexpressed tumor cells, class III β-tubulin overexpressed tumor cells, and multidrug-resistant tumor cells. Through exploring its mechanism, it was found that compound 65 could target colchicine binding sites, inhibit microtubules polymerization and spindle formation, and was more active than colchicine, and could overcome P-gp pump-mediated multi-drug resistance.

Zhu synthesized a class of 4-substituted coumarin H6 that has a strong ability to inhibit tumor cell proliferation with the IC50 range between 7 and 47 nM, and it also has a significant ability to inhibit tumor growth in paclitaxel-resistant tumor models. The mechanism is related to target colchicine binding site of β-tubulin. H6 is a powerful microtubule inhibitor. In order to solve its hydrophobicity limitation, Zhu prepared H6/MPEG2kPCL2k micelles by a simple thin-film hydration method, which significantly improve its solubility, reduce its toxicity and extend the half-life of the drug without affecting the anti-cancer property of this derivative (35).

Dahong tested the anti-proliferative activity of Ferulin C against breast cancer cells *in vitro* and *in vivo*. *In vitro*, the furan coumarin core of Ferulin C is in contact with β-tubulin through colchicine binding sites, thereby inhibiting tubulin aggregation. Ferulin C could markedly suppress the tubulin polymerization with IC50 of 9.2 μM and Colchicine (IC50 = 1.8 μM) was used as the reference compound. Further research suggested that Ferulin C only affected the structure of microtubules and had not effect on the expression of tubulin. Ferulin C induced microtubule instability, and subsequently activated p21 and inhibited PAK1. In breast cancer cells, elevated expression of PAK1 indicated poor survival and p21 was associated with better survival. Ferulin C induced G1/S cell cycle arrest *via* p21Cip1/Waf1-CDK2 signaling pathway. Dahong established a breast cancer cells xenograft model and evaluate antitumor activity of Ferulin C (low dose, 25 mg/kg; median dose, 50 mg/kg; high dose, 100 mg/kg) *in vivo*. Ferulin C could significantly inhibit the growth of xenograft breast cancer cells and the antitumor mechanism was significantly consistent with *in vitro* experiments (21).

### Reactive Oxygen Species Regulation

ROS is a type of natural by-products produced during cell metabolisms, such as superoxide anion, hydrogen peroxide, and peroxide hydrogen root, hydroxyl radical. In normal cells, medium and high concentrations of ROS are believed to be closely related to apoptosis caused by the cellular stress response, while low concentrations of ROS have a wide range of physiological significance, affecting some signaling pathways and activating transcription factors, and promoting cell proliferation and differentiation. In tumor cells, due to excessive metabolic activity and impaired mitochondrial function, ROS levels are much higher. In addition, ROS is required for tumorigenesis, tumor cell survival, proliferation, and metastasis (36). ROS mediates activation of transcription factor NF-κB, which promotes proliferation and metastasis of tumor cells (37).

The intracellular ROS levels of pancreatic cancer cells treated with or without esculetin were stained with DCFH-DA and detected by flow cytometry. Intracellular ROS and protein levels of the ROS-dependent transcription factor NF-κB decreased with time-dependent manner after esculetin exposure (28).

On the other hand, excessive ROS production in cells can induce apoptosis. The ability of ROS to cause severe cell damage and cell death has been used as a way to kill cancer cells. Han evaluated the production of intracellular ROS by DCFH-DA staining. Compared with the control group, the production of ROS in human cervical cancer cells treated with Shikonin derivative PMMB232 was significantly increased (30).

Glutathione is a natural antioxidant that neutralizes harmful ROS produced in normal cells, and its consumption plays a central role in cell death. 7,8-diacetoxy-3-(4-methylsulfonyl phenyl) coumarin (compound 5f) synthesized by Musa can consume glutathione, leading to excessive accumulation of ROS in prostate cancer cells and inducing apoptosis caused by oxidative stress (29).

Esculetin can induce the generation and accumulation of ROS in human throat epidermoid cancer cells, and also block cell cycle in the G1/S phase, thus inducing and promoting cell apoptosis (38).

Copper is found to be elevated in several types of cancer and involved in cancer growth, angiogenesis and metastasis, which has made it a potential target for developing anticancer therapeutics. Saman synthesized a coumarin-based copper chelator, di(2-picolyl)amine-3(bromoacetyl)coumarin hybrid molecule (ligand-L), and tested the anticancer activity of ligand-L in rat model of diethylnitrosamine induced hepatocellular carcinoma, including *ex vivo* and *in vivo* experiments. The *in vitro* suggested that ligand-L(25 μM) interacts with cellular copper and generates ROS which causes oxidative DNA damage and cell death of hepatocellular carcinoma cells. *In vivo* experiments, ligand-L(250 mg kg^−1^ body weight) significantly suppressed the progression of diethylnitrosamine -induced liver cancer by producing copper-ligand-L and thereby produces ROS, and showed not toxic to normal rat liver tissue. The *in vivo* findings revealed that ligand-L shows a chemopreventive effect as it decreased levels of serum liver markers and alpha fetoprotein, and improved liver architecture (39).

### Inhibition of Tumor Angiogenesis

Angiogenesis is the process of forming and recruiting new blood vessels from preexisting vascular system, which is necessary for solid tumor growth. Therefore, targeting tumor angiogenesis is one of the most critical anti-tumor strategies.

Naipeng tested the effects of three triphenylethylene-coumarin hybrids (TCHs) on endothelial cell migration and angiogenesis induced by breast cancer cells *in vivo* and *in vitro*. Among them, TCH-5C (5 and 10 μM) showed high anti-proliferative activity and low cytotoxicity in human umbilical vein endothelial cells. In addition, TCH-5C (2.5 μM) can inhibit the formation of microtubules in human umbilical vein endothelial cells, destroy cytoskeleton, and inhibit the migration of human umbilical vein endothelial cells. TCH-5C (5 μM) also increased the expression of cyclin-dependent kinase inhibitor P21 and decreased the level of cyclin B1, which induces cell cycle arrest and inhibits the proliferation of human umbilical vein endothelial cells. *In vitro*, TCH-5C (5 μM) reduced the production and secretion of VEGF in breast cancer cells and directly inhibited the formation and migration of endothelial cell tubes induced by breast cancer cells. *In vivo*, tumors in the nude mouse model treated with TCH-5C were significantly smaller in size and weight than those in the untreated control group. In the nude mouse model, TCH-5C also reduced serum VEGF levels, suggesting that TCH-5C inhibits tumor progression by reducing VEGF-induced angiogenesis (40).

da Cruz evaluated the toxicity and antitumor mechanism of 7-isopentenyloxycoumarin (UMB-07) on Ehrlich ascites carcinoma model. The results showed that the LD50 value of UMB-07 was about 1000 mg/kg, and at the concentration of 50 mg/kg, UMB-07 had significant anti-tumor activity *in vivo*, indicating that UMB-07 was a good candidate anti-tumor drug worthy of further study. The main antitumor mechanism of UMB-07 is the inhibition of tumor angiogenesis by reducing chemokine CCL2, and CCL2 is important to tumor neovascularization by inducing increased VEGF in tumor microenvironment. However, this coumarin also induced gastrointestinal toxicity and produced a reduction on nutrient absorption, which caused a reduction in the weight of animals and an increase on feed intake (41).

A series of coumarin derivatives incorporating different functional groups were synthesized and tested for their antiproliferative activity against breast cancer and prostate cancer cells. They all revealed more cytotoxic activity on breast cancer cells than the reference standard staurosporine (IC50 = 8.81 µM) with the IC50 values ranged from 1.24 to 8.68 µM. Eman further explored the antitumor mechanism of 2-(4-methyl-2-oxo-2H-chromen-7-yloxy)-N’-benzylacetohydrazide(4a), which is the most cytotoxic of all. 4a targeted VEGFR-2 and reduced its activity, thereby inhibiting tumor angiogenesis. 4a also induced preG1 apoptosis and prevented cell growth in G2/M phase and activated caspase-9. However, none of these derivatives showed significant cytotoxic activity on prostate cancer cells (42).

Signal transducer and activator of transcription-3 (STAT3) gene is an oncogene and is overexpressed in many tumor types. STAT3 expression and phosphorylation increased with malignant progression of laryngeal cancer. STAT3 mediates angiogenesis in laryngeal cancer, and inhibition of the JAK-2/Stat-3 signaling pathway significantly inhibits invasion *in vitro* and angiogenesis of laryngeal cancer. Esculetin inhibits migration and invasion of laryngeal cancer by inhibiting STAT3 phosphorylation and preventing STAT3 transport into the nucleus. In addition, Colivelin can reverse the anticancer effect of Esculetin on laryngeal cancer by activating STAT3, further proving that Esculetin functions by inhibiting STAT3 phosphorylation (38).

### Other Mechanisms

Nrf2, which is strictly regulated by KEAP1, is an important anti-tumor factor and a major regulator of antioxidant reactions. It can bind to antioxidant reaction elements in the promoters of antioxidant proteins and activate the coding of these proteins (43). In tumor cells, KEAP1 sequesters Nrf2 in the cytoplasm and promotes its ubiquitination and proteasomes degradation (44). In order to detect whether esculetin affects the regulation of the antioxidant reaction elements pathway, Arora treated pancreatic cancer cells with esculetin and detected the interaction between Nrf2 and KEAP1, and found that esculetin could directly bind to KEAP1, then destroying the interaction of Nrf2-KEAP1, releasing Nrf2 from the inhibition of KEAP1, increasing the amount of phosphorylation of Nrf2 in cells and increasing the expression of NAD(P)H quinone oxidoreductase-1 (NQO1), the direct target of Nrf2 (28).

NQO1 is a very important enzyme in human cells, whose functions include xenogenic detoxification, superoxide removal, regulation of p53 proteasome, degradation and maintenance of endogenous antioxidants (45). NQO1 is highly expressed in many tumors and play an important role for tumor growth and migration. Studies have shown that NQO1 may be a target for cancer therapy, and inhibition of NQO1 may improve the efficacy of antitumor chemotherapy. By means of NQO1 activity assay, Khunluck studied the inhibitory effects of 21 natural compounds on NQO1 and found that coumarin compounds (coumarin, aesculetin, umbelliferone, and scopoletin) had the stronger inhibitory effect on NQO1, so these componuds could be used as non-competitive inhibitors of NQO1. The inhibitory rate of scopolamine on NQO1 (77.38%) was the highest. In cholangiocarcinoma cells, scopoletin and umbelliferone showed a strong inhibitory effect on NQO1, and scopoletin also showed strong cytotoxicity on cholangiocarcinoma cells. In order to explore whether NQO1 was involved in the cytotoxicity of scopoletin to cholangiocarcinoma cells, Khunluck also tested the cytotoxicity of scopoletin to cholangiocarcinoma cells that had been introduced with NQO1 siRNA and found that the cytotoxicity was reduced. Scopoletin can also inhibit the migration of bile duct cancer cells by reducing the mRNA ratio of the migration-related gene MMP9/TIMP1 (46).

Aldehyde dehydrogenase (ALDH), which catalyzes the oxidation of acetaldehyde and other aliphatic aldehydes, is a key enzyme in the ethanol metabolic pathway and an important molecule of antioxidant stress. It is also one of the common markers of normal stem cells and cancer stem cells. In breast cancer cells, highly active ALDH leads to breast cancer stem cell characterization by upregulating Notch-1 and epithelial-mesenchymal markers. Activation of the Notch-1 signal plays an important role in the self-renewal, proliferation, and apoptosis of tumor cells. Abnormal activation of Notch1 is relatively common in many tumor types. Deeksha examined the functional and clinical significance of ALDH expression by immunohistochemical measurements in breast cancer tissue. ALDH expression is significantly higher in higher grade breast tumors tissues (grade II and III) than in normal breast tissue. Injection of breast cancer stem cell (ALDH+ and CD44+/CD22−) cells resulted in invasive tumor growth in athymic mice compared with ALDH− cells. ALDH+ and CD44+/CD22− tumors grow rapidly and are larger than the slow-growing and smaller ALDH− tumors. On the molecular level, the ALDH+ tumor has higher expression of Notch-1 and EMT markers than ALDH− tumor. Oral administration of the naturally occurring Psoralidin (25mg/kg of body weight) highly inhibited the growth of ALDH+ and ALDH− tumors on xenograft models. In addition, Psoralidin inhibited Notch-1 mediated EMT activation in ALDH+ and ALDH− tumors. Therefore, Psoralidin may prevent the incidence and metastasis of breast cancer by significantly inhibiting Notch-1 in breast cancer cells (47).

Monocarboxylate transporter (MCT) transports pyruvate and lactic acid accumulated during glycolysis from tumor cells to avoid cytoplasmic acidification, which may lead to apoptosis. There are 14 known isoforms of MCTs, and MCT1-4 is responsible for transporting these carboxylate. They are also associated with the influx and outflow of lactate, which can produce energy in cancer-related stromal fibroblasts and epithelial cancer cells. Elevated MCT1 expression has been identified in a large number of cancers, so this transporter may be a target for broad-spectrum cancer therapy. Gurrapu (48) synthesized a series of N, N-dialalkyl carboxyl coumarins as MCT1 inhibitors, and tested cytotoxicity of these coumarins on MCT1 and MCT4 expression cell lines *in vitro*. The lead compound 4a (7-(dibenzylamino-2-oxo-2h-Chromene-3-carboxylic acid) was highly effective in inhibiting glioblastoma that was predominantly MCT1 expression but did not show any activity in triple-negative breast cancer that was MCT4 expression.

HIF-1 is a major activated transcription factor under hypoxia conditions. Its activity is mainly determined by HIF-1α. Members of the HIF-1α family are key regulators of glycolysis. HIF-1α is widely overexpressed in human cancers to prevent tumor apoptosis due to oxygen depletion. Activation and stabilization of the HIF-1α signaling pathway induce the expression of many target genes involved in tumor cell growth, metabolism, and invasion. The shikonin derivative PMMB232 synthesized by Han et al. can bind to HIF-1α, thereby promoting HIF-1α degradation, impairing glycolysis in cancer cells and promoting apoptosis. PMMB232 can also up-regulate the expression of E-cadherin, enhance cell adhesion, and inhibit the migration and metastasis of tumor cells (30).

AKR1B10 (Aldo-Keto Reductase 1B10), which is highly expressed in normal gastrointestinal epithelial tissues, but low or no expression in other normal tissues, is an NADPH-dependent reductase whose function is mainly to catalyze the reduction of various carbonyl compounds. AKR1B10 has also been found to be overexpressed in human non-small cell carcinoma, liver cancer, breast cancer, oral squamous cell carcinoma and pancreatic cancer, and is involved in the occurrence, development and survival of tumor cells through a variety of mechanisms, including cytotoxic reactive carbonyl compounds Detoxification, enhance the migration and proliferation potential of tumor cells, regulate cellular fatty acid synthesis and lipid metabolism, and participate in the acquisition of multidrug resistance in tumor cells. HMCB (7-hydroxy-2-(4-methoxyphenylimino)-2H-chromene-3-carboxylic acid benzylamide) is the most effective AKR1B10 inhibitor currently but does not have a high selectivity. Satoshi et al. took HMCB as a starting material to synthesize a series of coumarin derivatives, which have stronger selectivity and inhibition for AKR1B10. These coumarin derivatives significantly inhibited the migration and proliferation of lung cancer cells, increased the cisplatin sensitivity of cisplatin-resistant lung cancer cells, and inhibited the metastasis and invasion potential of cisplatin-resistant lung cancer cells (49). Awale et al. suggested that AKR1B10 may also be related to the tolerance of pancreatic cancer to nutritional deficiency. Human pancreatic cancer cells are resistant to nutritional starvation by switching energy metabolism, allowing them to survive in tumor microenvironments where blood vessels are scarce. Awale synthesized a series of coumarin derivatives based on HMCB structure and evaluated their inhibition against the resistance to nutritional starvation of human pancreatic cancer cells. 7-hydroxy-2-oxo-2H-chromene-3-carboxylic acid (3-phenylpropyl)amide (compound 2c) showed highly selective cytotoxicity on human pancreatic cancer with a PC50 value of 0.44 mM but no toxicity on conventional nutrient-rich media (50).

## Discussion

**Figure 2:**
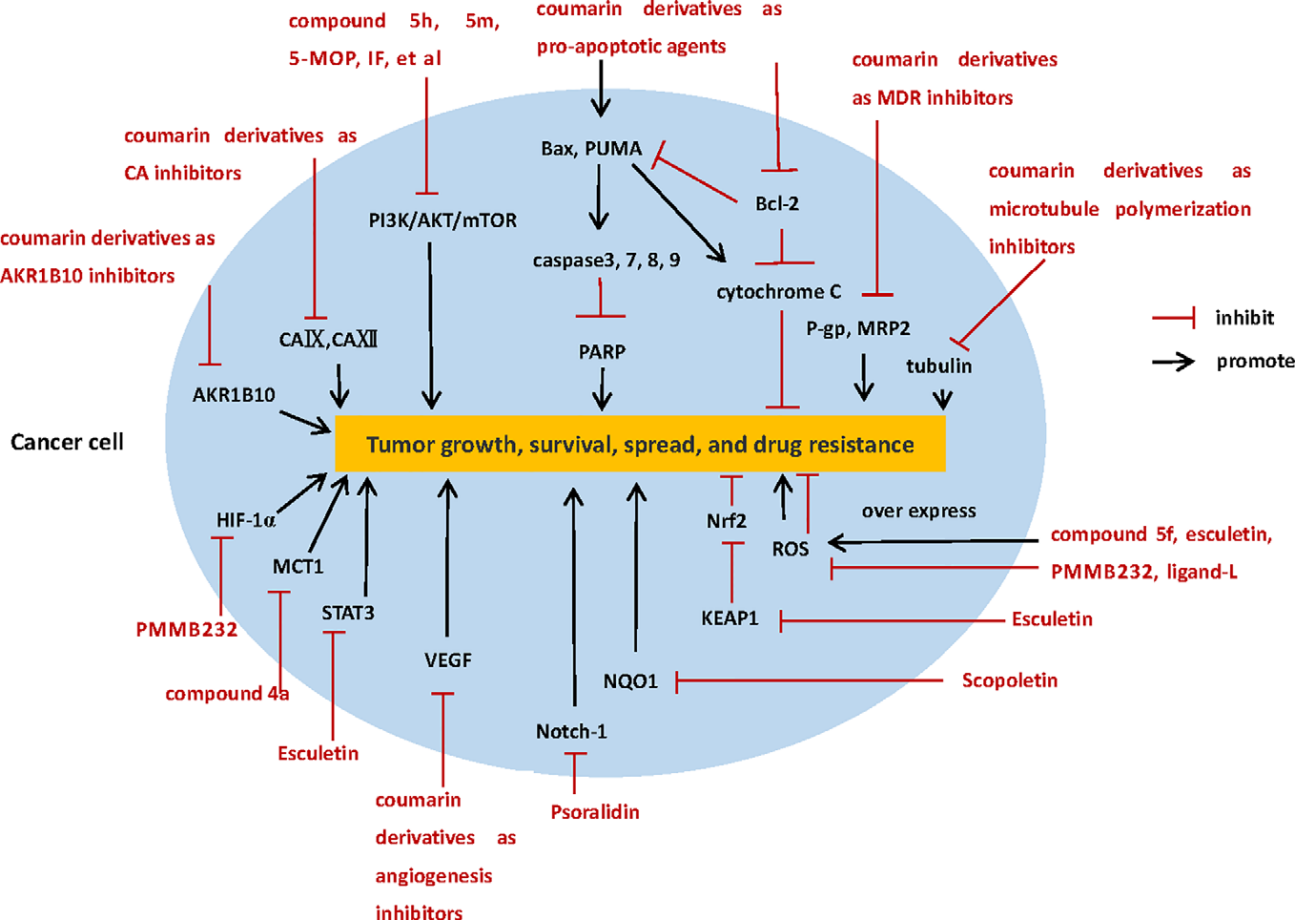
The diverse anti-tumor mechanisms of coumarin derivatives.

Coumarin compounds can inhibit the growth, proliferation and metastasis of various tumor cells through a variety of mechanisms, including inhibition of carbonic anhydrase, PI3K/AKT/mTOR signaling pathway, microtubule polymerization, angiogenesis, monocarboxylate transporters, hypoxia-inducible factor-1; acting on apoptosis proteins and inhibiting tumor multidrug resistance, regulation ROS, and so on (**Figure 2**). Whether it is a natural coumarin extracted from various plants or new coumarin derivative synthesized by modification of the basic structure of coumarin, *in vitro* experiments, could inhibit the growth and proliferation of tumor cells at low concentration, and did not produce poisonousness to the normal cells, which proved that coumarins are a class of promising anti-tumor drugs with high selectivity.

However, there are still many problems to be solved before coumarins can be used clinically. First, the current studies of coumarins as new antitumor drugs still in a primary stage, many of the studies on the anti-tumor mechanisms of coumarins have not been studied deeply, and stopped at a relatively simple mechanism. Second, most studies are limited in the *in vitro* experiments, lack of *in vivo* evidence to support, so the antitumor potential and security of coumarins *in vivo* is not clear, which needs further research. Third, coumarin compounds are fat-soluble, and have poor solubility in water, in order to enhance their bioavailability, may need to improve the concentration or the use of cosolvent, carrier system to increase their solubility and bioavailability, or through the structure modification or the introduction of new functional groups, can give coumarin compounds new performance. However, these measures may affect the antitumor activity and safety of coumarin compounds *in vivo* and *in vitro*.

On the whole, numerous *in vitro* and *in vivo* experiments proved coumarins are indeed a kind of potential new antitumor drugs, which have selective cytotoxicity and diverse anti-tumor mechanisms (**Table 1**), but there are still problems that most of these studies are not in-depth or to explore the relationship between mechanisms, lack of *in vivo* experiments results in its unknown of security and drug metabolism *in vivo*, and low solubility in water limits its bioavailability, however, we believe that the modifications based on the structure of coumarin can solve these problems in the near future. We still have high hopes for coumarin compounds, because the structure of coumarin determines its many biological pharmacological activities, and the heterocyclic structure of coumarin is easy to bind to a variety of target proteins. The 2H-chromen-2-one ring can interact with different biological antigens because of its aromatic, planar, and lipophilic. In addition, the lactone group of coumarin enables the molecule to form strong polar bonds, such as hydrogen bonds and acetylated protein targets (51). Researchers may be able to link the antitumor mechanisms, water solubility with structure and group, by modifying the structure to produce coumarin derivatives with higher selectivity, less side effects, and higher bioavailability. We look forward to more studies on the anti-tumor mechanisms of coumarins, especially *in vivo* experiments and preclinical studies, to evaluate the efficacy, safety, and pharmacokinetic properties of coumarins, so as to accelerate the development of coumarins as new anti-tumor drugs and provide a new strategy for clinical cancer treatment.

**Table 1:** Summary of the mechanisms of coumarins as antitumor drugs.

Coumarins	Test type	Effective concentration	Mechanisms
Compound 8i (5)		CA IX: 45.5 nM CA XII: 596.6 nM	selective inhibitory activities to CA IX and CA XII
Compound 5p and 5n (6)		CA IX: 144.6 nM and 71.5 nM	
1-aryl and 2-aryl-substituted Coumarin derivatives (7)		CA IX: 9.6 nM CA XII: 5.8 nM and 25.2 nM	
4-sulfamoylphenyl/sulfocoumarin benzamides (8)	*In vitro*	CA IX: 99.4–4092.5 nM CA XII: 30.6–7461.5 nM	
dihydroartemisinin-coumarin hybrids (9)		CA IX: 0.17 μM CA XII: 0.17 μM	
2-thioxocoumarine derivative 15 and 16 (10)		0.004–0.027 μM	
Compound 5h and 5m (15)		18.1–32.6 μM and 29.3-42.1 μM	inhibition of the expression or phosphorylation of PI3K, Akt, and mTOR
5-methoxypsoralen (16)		75 μM	
Isofraxidin (17)		40 μM	
TCHD (18)	*In vitro*	75–125 μg/ml	
Imperatorin (19)		75 μM	
3-benzyl coumarin deca-B-ring derivative (20) Ferulin C (21)		53 nM 20 μM	
Ferulin C (21) Xanthyoxylin and 6, 7, 8-trimethoxyl-coumarin (24)		20 μM 0.1 μg/ml	up-regulate the expression of pro-apoptotic proteins, and down-regulate the expression of anti-apoptotic proteins
Isofraxidin (17)		10 μg/ml	
TCHD (18)		75–125 μg/ml	
8-methoxy coumarins hybridize with three bioactive moieties (25)	*In vitro*	0.65–0.93 μM	
PulchrinA (26)		22 μM	
A nitro-coumarin derivate (27)		40 μM	
Esculetin (28)		100 μM	up-regulate the expression of pro-apoptotic proteins, and down-regulate the expression of anti-apoptotic proteins; induce the generation and accumulation of ROS overly or decrease the intracellular ROS level
Compound 5f (29)		26.43 μM	
PMMB232 (30) Esculetin (38) Ligand-L (39)	*In vitro* *In vitro* *In vitro*	3.25±0.35 μM 10 μM 250 mg/kg	
Mansorin-II; (31) Coumarin derivatives (32)	*In vitro*	0.74–36 µM	interfer with the efflux activity of P-gp or MRP2
Conferdione and Samarcand (33)		22.75 ± 3.88 μM and >50 µM	
Ferulin C (21) Compound 65 (34)	*In vitro* *In vitro*	25 mg/kg 3.5–31.9 nM	target colchicine binding sites, inhibit microtubules polymerization
4-substituted coumarin H6 (35)		7-47 nM	
TCH-5C (40) 7-isopentenyloxycoumarin (41) Compound 4a (42)	*In vitro* *In vitro* *In vitro*	3 mg/kg 50 mg/kg 1.24–8.68 µM	reduce VEGF-induced angiogenesis
Esculetin (38)	*In vitro*	10 μM	inhibit STAT3 phosphorylation
Esculetin (28)	*In vitro*	100 µM	increase the amount of anti-tumor factor Nrf2
Scopoletin (46)	*In vitro*	0.88 mM	inhibitory effect on NQO1
Psoralidin (47)	*In vitro*	25 mg/kg	inhibit Notch-1 in breast cancer cells
Compound 4a (48)	*In vitro*	0.09 ± 0.01µM	inhibition of the expression of MCT1
PMMB232 (30)	*In vitro*	4µM	promote HIF-1α degradation; up-regulate the expression of E-cadherin
Coumarin derivatives based on HMCB (49) compound 2c (50)	*In vitro*	4.1 ± 0.3nM and 3.4 ± 0.1nM 0.44 mM	inhibition for AKR1B10

## Author Contributions

YW, JX, and YL wrote the first draft of the manuscript. JX, YL, and YZ organized the literature search. GW supervised the study and edited the manuscript. YW, JX, YL, and YZ wrote sections of the manuscript. All authors contributed to the article and approved the submitted version.

## Funding

This work was supported by the Natural Science Foundation of Hunan Province (Project Number: 2019JJ40365) and the Innovation and Entrepreneurship Training Program for College students of 2020 grade (Project Number: S2020105330352).

## Conflict of Interest

The authors declare that the research was conducted in the absence of any commercial or financial relationships that could be construed as a potential conflict of interest.

## Abbreviations

CA, Carbonic anhydrase; MDR, Multiple drug resistence; HIF, Hypoxia-inducible transcription factor; TCHD, The total coumarins of Hedyotis diffusa; PARP, Poly-ADP-ribose polymerase; Bcl-2, B cell lymphoma-2; P-gp, P-glycoprotein; ROS, Reactive oxygen species; NQO1, NAD(P)H quinone oxidoreductase-1; ALDH, Aldehyde dehydrogenase; TCHs, Triphenylethylene-coumarin hybrids; STAT3, Signal transducer and activator of transcription-3; MCT, Monocarboxylate transporter; AKR1B10, Aldo-Keto Reductase 1B10.
